# Supplementary material for: CD8+ T cell landscape in Indigenous and non-Indigenous people restricted by influenza mortality-associated HLA-A*24:02 allomorph
Source: Nat Commun. 2021 May 18;12:2931. doi: 10.1038/s41467-021-23212-x (PMC8132304; doi:10.1038/s41467-021-23212-x)
Supplement: Supplementary file 2 — Supplementary Information [file 41467_2021_23212_MOESM2_ESM.pdf]

SUPPLEMENTARY INFORMATION

CD8$^+$ T-cell landscape in Indigenous and non-Indigenous people restricted by influenza mortality-associated HLA-A*24:02 allomorph

Luca Hensen[1*], Patricia T. Illing[2*], E. Bridie Clemens[1*], Thi H.O. Nguyen[1], Marios Koutsakos[1], Carolien E. van de Sandt[1,3], Nicole A. Mifsud[2], Andrea T. Nguyen[2], Christopher Szeto[2], Brendon Y. Chua[1], Hanim Halim[2], Simone Rizzetto[4], Fabio Luciani[4], Liyen Loh[1], Emma J. Grant[1,2], Phillipa M. Saunders[1], Andrew G Brooks[1], Steve Rockman[1,5], Tom C. Kotsimbos[6,7], Allen C. Cheng[8,9], Michael Richards[10], Glen P. Westall[11], Linda M. Wakim[1], Thomas Loudovaris[12], Stuart I. Mannering[12], Michael Elliott[13,14], Stuart G. Tangye[15,16], David C Jackson[1], Katie L Flanagan[17,18,19,20], Jamie Rossjohn[2,21,22], Stephanie Gras[2,22,23], Jane Davies[24], Adrian Miller[25], Steven Y.C. Tong[10,24]†, Anthony W. Purcell[2]† and Katherine Kedzierska†[1#]

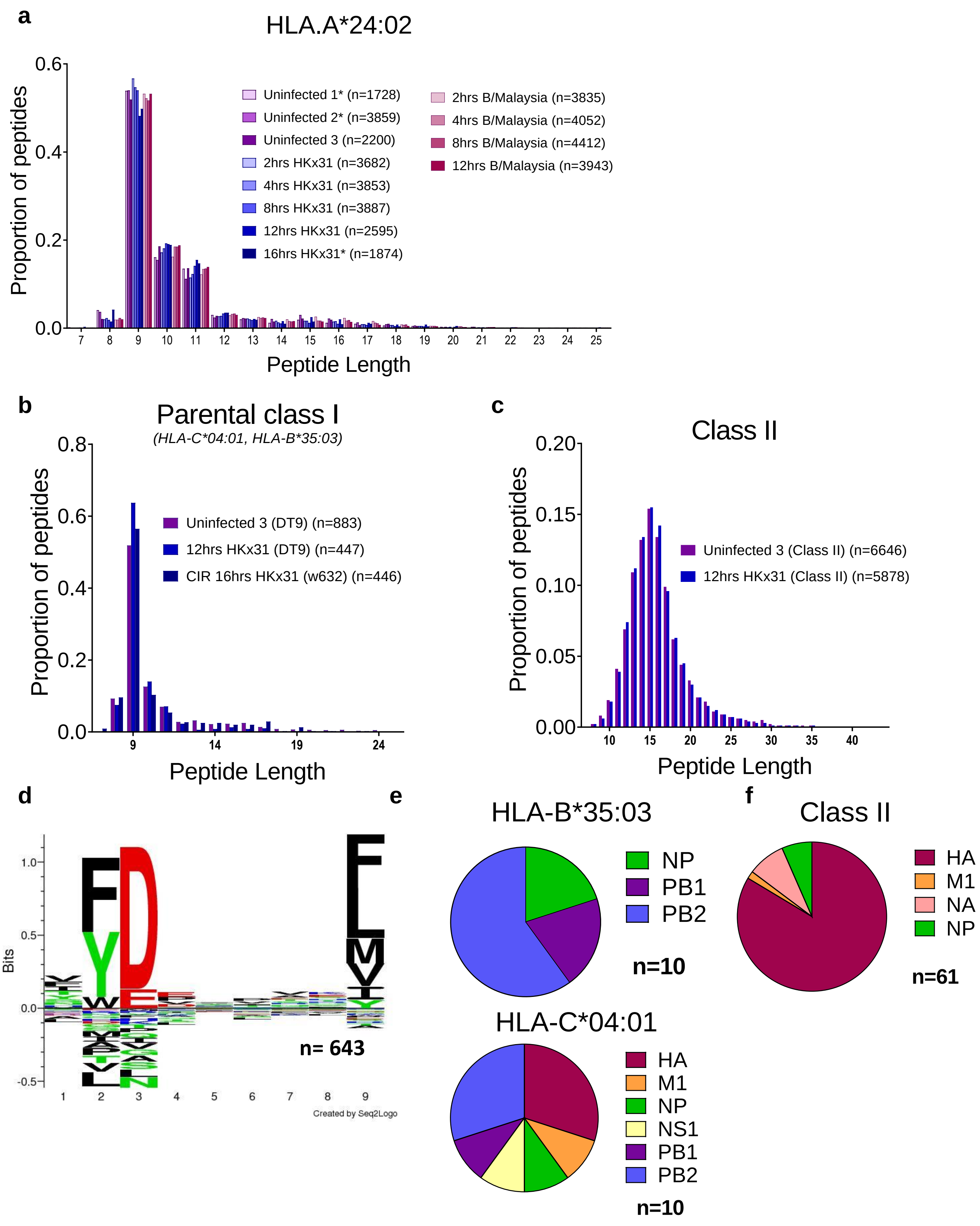

Supplementary Figure 1 Hensen*, Illing*, Clemens* *et al*

Supplementary Figure 1. Further analysis of LC-MS/MS data sets. (a-c) Peptide length distributions identified at a 5% FDR in each HLA isolation performed shown as a proportion of the peptides identified in the data set (n=number of peptides in the data set). For uninfected and HKx31 samples, data shown are based on searches against the human proteome, HKx31 proteome and 6 reading frame HKx31 genome translation. For B/Malaysia samples, data are based on searches against the human proteome, B/Malaysia proteome and 6 reading frame B/Malaysia genome translation. **(a)** Peptides from 12 data sets isolated from CIR.A24 using W6/32 (pan class I antibody) at different timepoints of infection with IAV or IBV (or uninfected). Asterisks represent data sets where DT9 was not used to deplete HLA-C*04:01 prior to W6/32 isolation and may contain increased levels of HLA-C*04:01 ligands. All data sets contain low levels of peptides presented by HLA-B*35:03 of CIR. **(b)** 3 data sets containing peptides isolated from the endogenous HLA class I of the CIR cell line, either through isolation from non-transfected CIR using W6/32 (CIR 16hrs HKx31 (W6/32)), hence containing HLA-C*04:01 and HLA-B*35:03, or through specific isolation of HLA-C*04:01 from CIR.A24 using the DT9 antibody. **(c)** HLA class II peptide ligands from 2 data sets isolated from CIR.A24 cell line using LB3.1 (HLA-DR), SPV-L3 (HLA-DQ), and B721 (HLA-DP) antibodies. **(d)** The sequence logo generated from human-derived 9mer peptides isolated in (b) (non-redundant by sequence, 5% FDR), and filtered for peptides identified in HLA class II isolations (c). **(e,f)** Pie charts showing the distribution of IAV (HKx31) derived peptides across the viral proteome potentially bound to HLA-B*35:03 and HLA-C*04:01 (e) and HLA-II of CIR cells (f). Details of HLA isolation antibodies, influenza peptide sequences, their confidence of sequence assignment, and potential binding assignment to the HLA molecules expressed by CIR.A24 are provided in **Supplementary Data 1**.

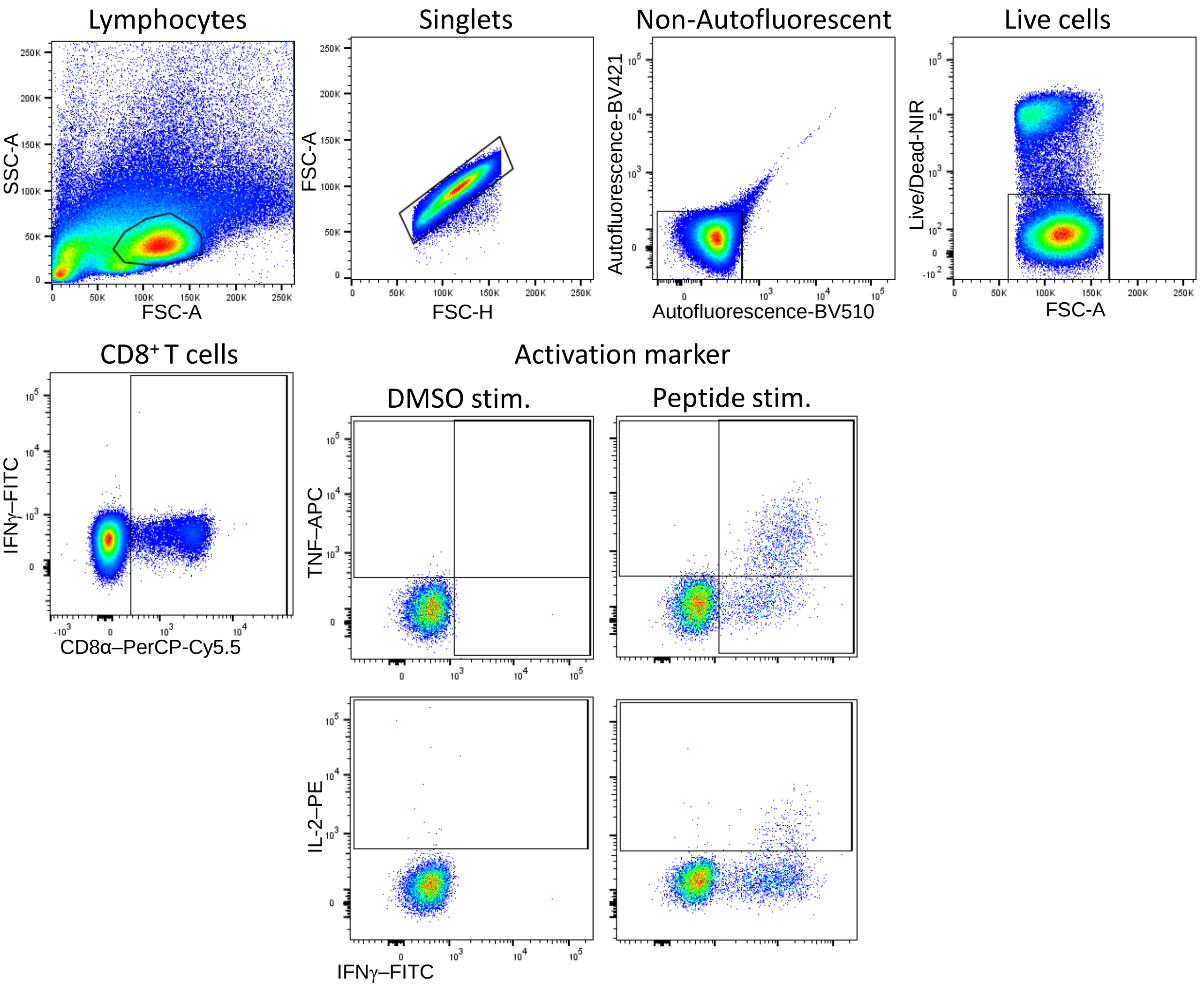

Supplementary Figure 2. Gating of mouse ICS results. Single cell suspensions were gated on live, lymphocytes without autofluorescence to assess expression of IFN-γ, TNF and IL-2 on CD8^{+} T cells after peptide stimulation.

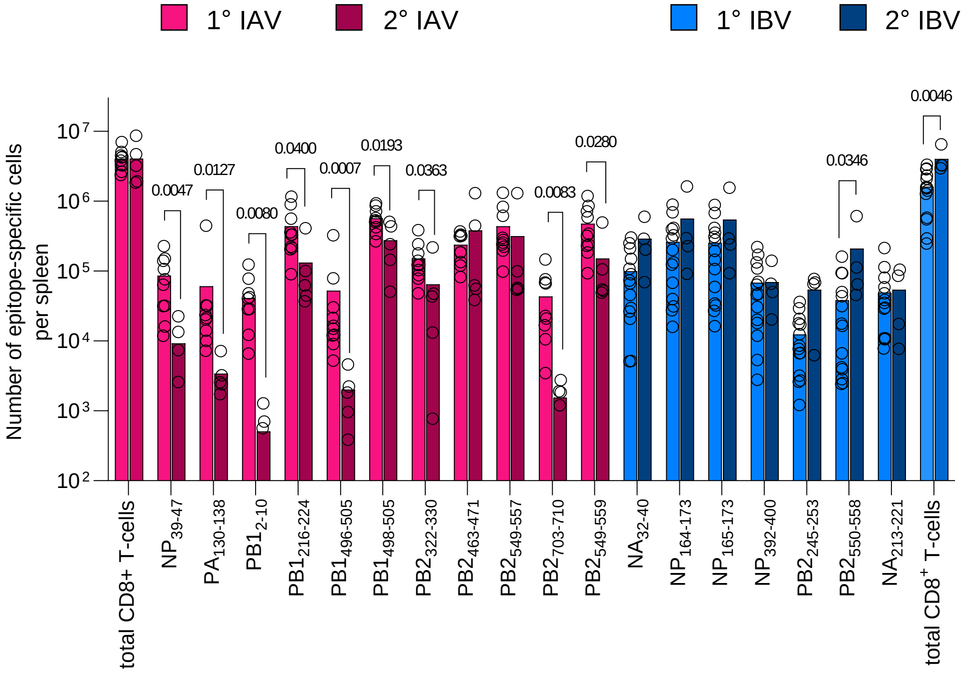

Supplementary Figure 3. Comparison of epitope-specific $CD8^+$ T-cells in primary and secondary IAV and IBV infection. Total number of epitope-specific $CD8^+$ T-cells calculated by the frequency of $IFN\gamma^+CD8^+$ T-cells in the INF-γ ICS assay multiplied by the total number of splenocytes per spleen (IAV primary n=10 mice from two independent experiments; IAV secondary n=5 mice from 1 experiment; IBV primary n=14 mice from three independent experiments; IBV secondary n=4 mice from 1 experiment). Statistical analysis was performed with a two-tailed Mann Whitney test. P values are indicated when <0.05.

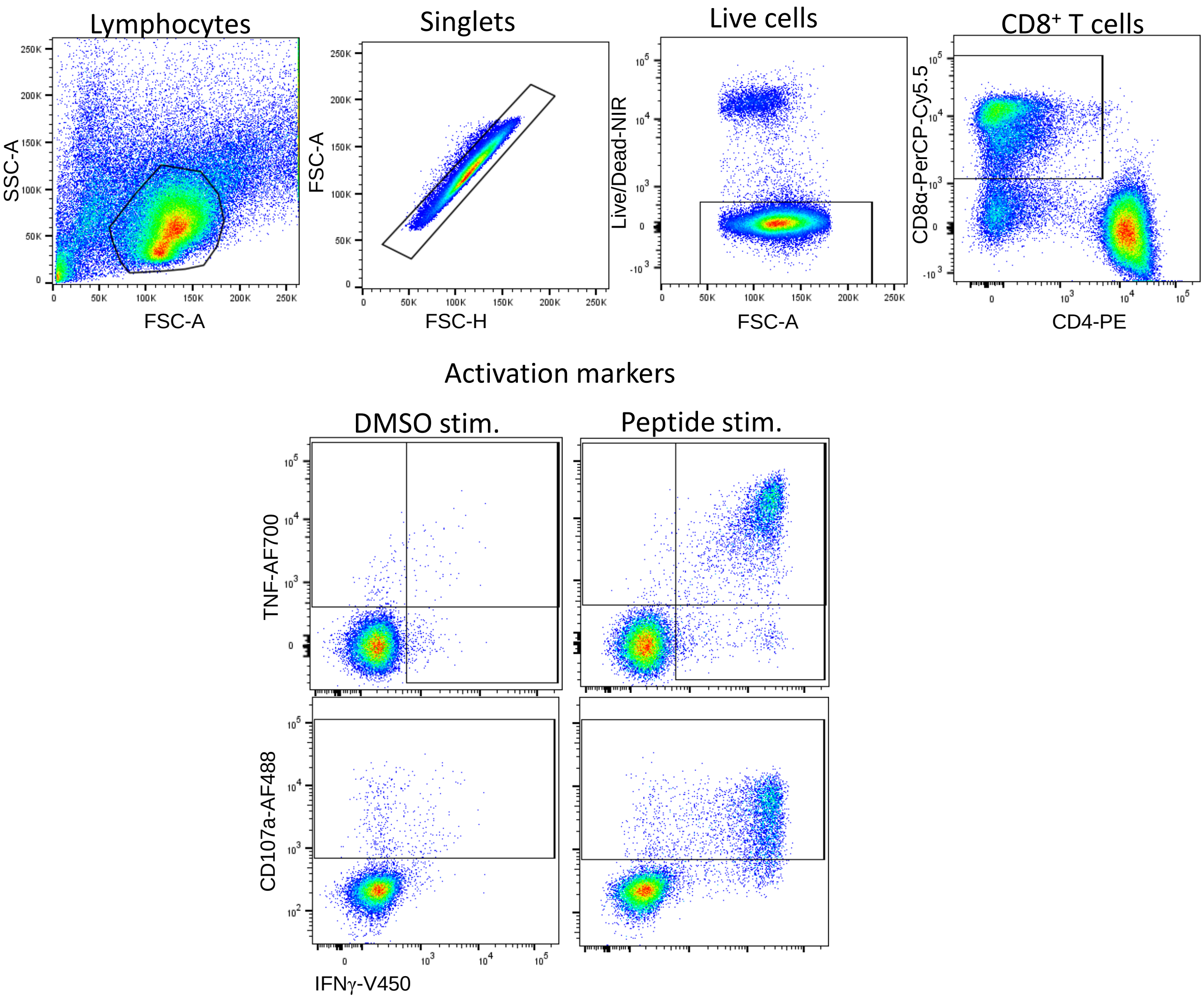

Supplementary Figure 4. Gating of human CD8^{+} T cell ICS. Single cell suspensions were gated on live, lymphocytes to assess expression of IFN-γ, TNF and CD107a on CD8^{+} T cells after peptide stimulation.

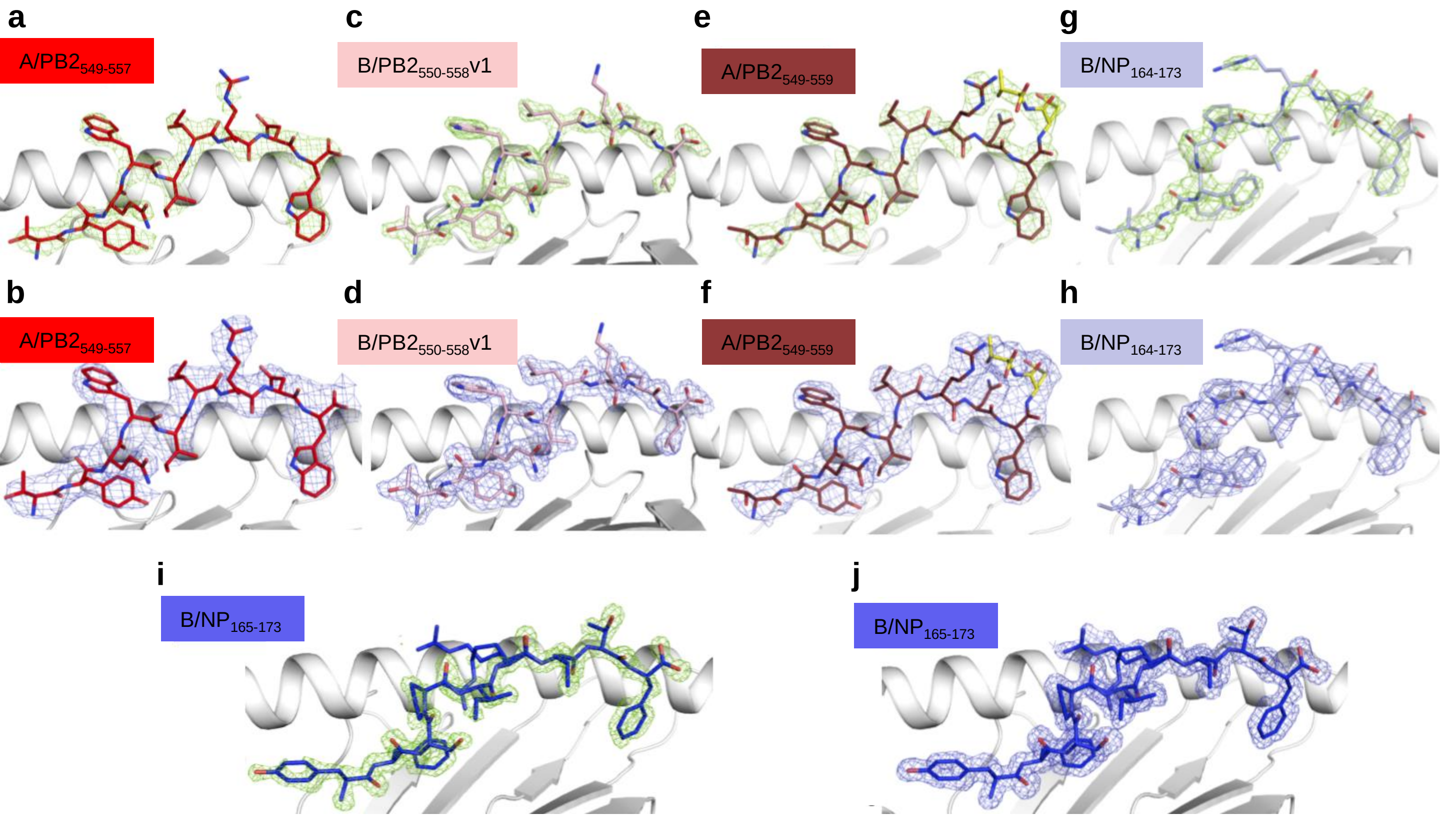

Supplementary Figure 5. Electron density maps of the peptide-HLA-A*24:02 structures. Peptide presentation in the antigen binding cleft of HLA-A*24:02 showing electron density omit maps (Fo-Fc) at 3.0σ (green) and electron density maps (2Fo-Fc) at 1.0σ (blue), for **a-d** and **i** and **e-h** and **j** respectively. The HLA-A*24:02 is represented as white cartoon, while the peptides are represented in sticks and coloured in red (A/$PB2_{549-557}$), pink (B/$PB2_{549-557}$v1), maroon (A/$PB2_{549-559}$), blue (B/$NP_{165-173}$), and light blue (B/$NP_{164-173}$).

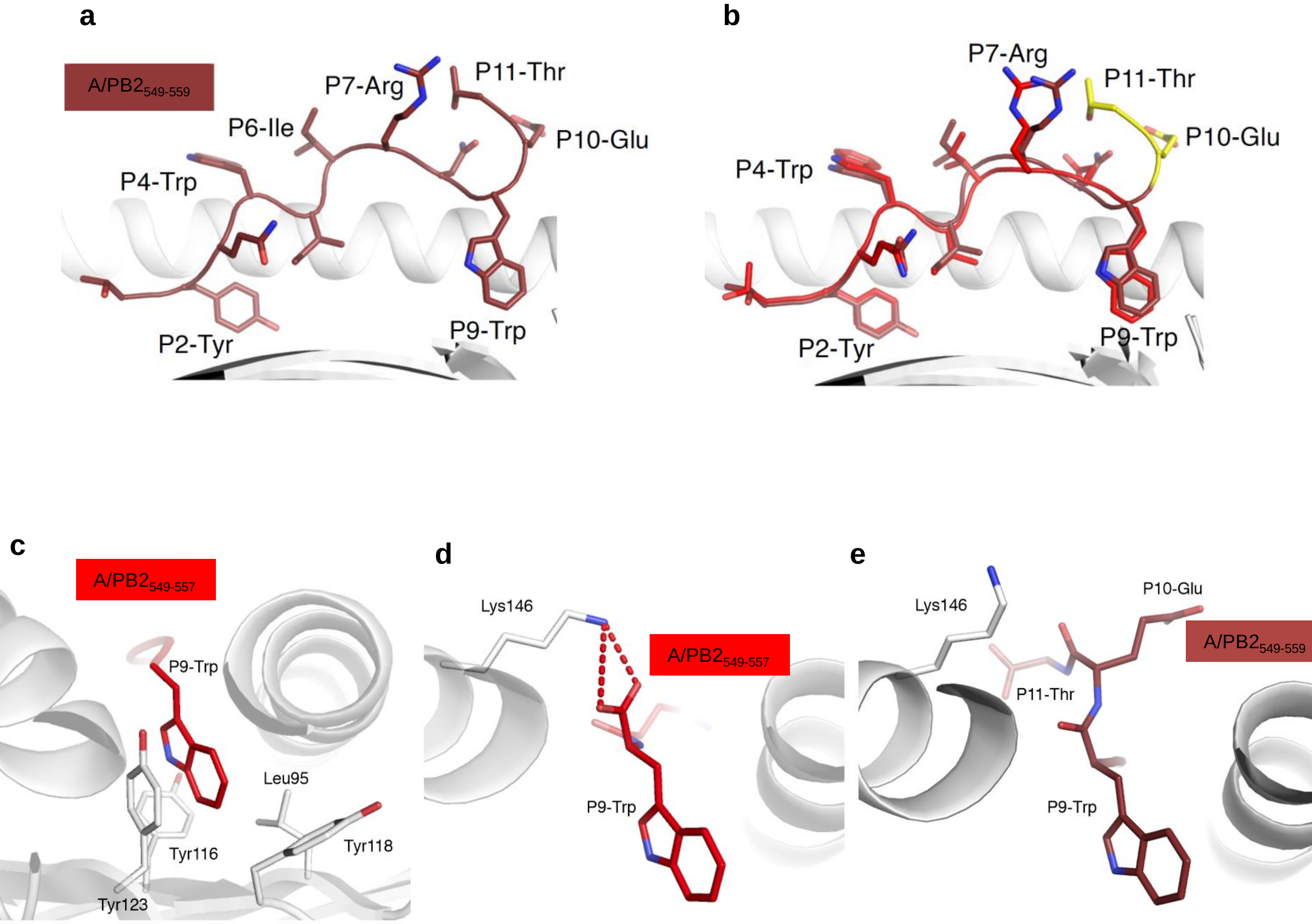

Supplementary Figure 6. Crystal structures of HLA-A*24:02 presenting the A/PB2$_{549\text{-}559}$ peptide. **(a)** Depiction of the PB2$_{549\text{-}559}$ peptide presented by HLA-A*24:02. **(b)** Overlay of A/PB2$_{549\text{-}557}$ (red) and PB2$_{549\text{-}559}$ (maroon) peptides presented by HLA-A*24:02. The two extra residues of the A/PB2$_{549\text{-}559}$ peptide are shown in yellow. **(c)** The P9-Trp of the A/PB2$_{549\text{-}557}$ peptide (red) is forming a network of interactions with Tyr at positions 116, 118 and 123 and Leu95 within the peptide-binding cleft. **(d)** Lys146 of the α2-helix of HLA-A*24:02 also interacts with the carboxylic group of the PΩ residue of the A/PB2$_{549\text{-}557}$ 9mer peptide (red) but **(e)** faces outside of the cleft and does not interact with the P9-Trp of the A/PB2$_{549\text{-}559}$ 11mer peptide (maroon).

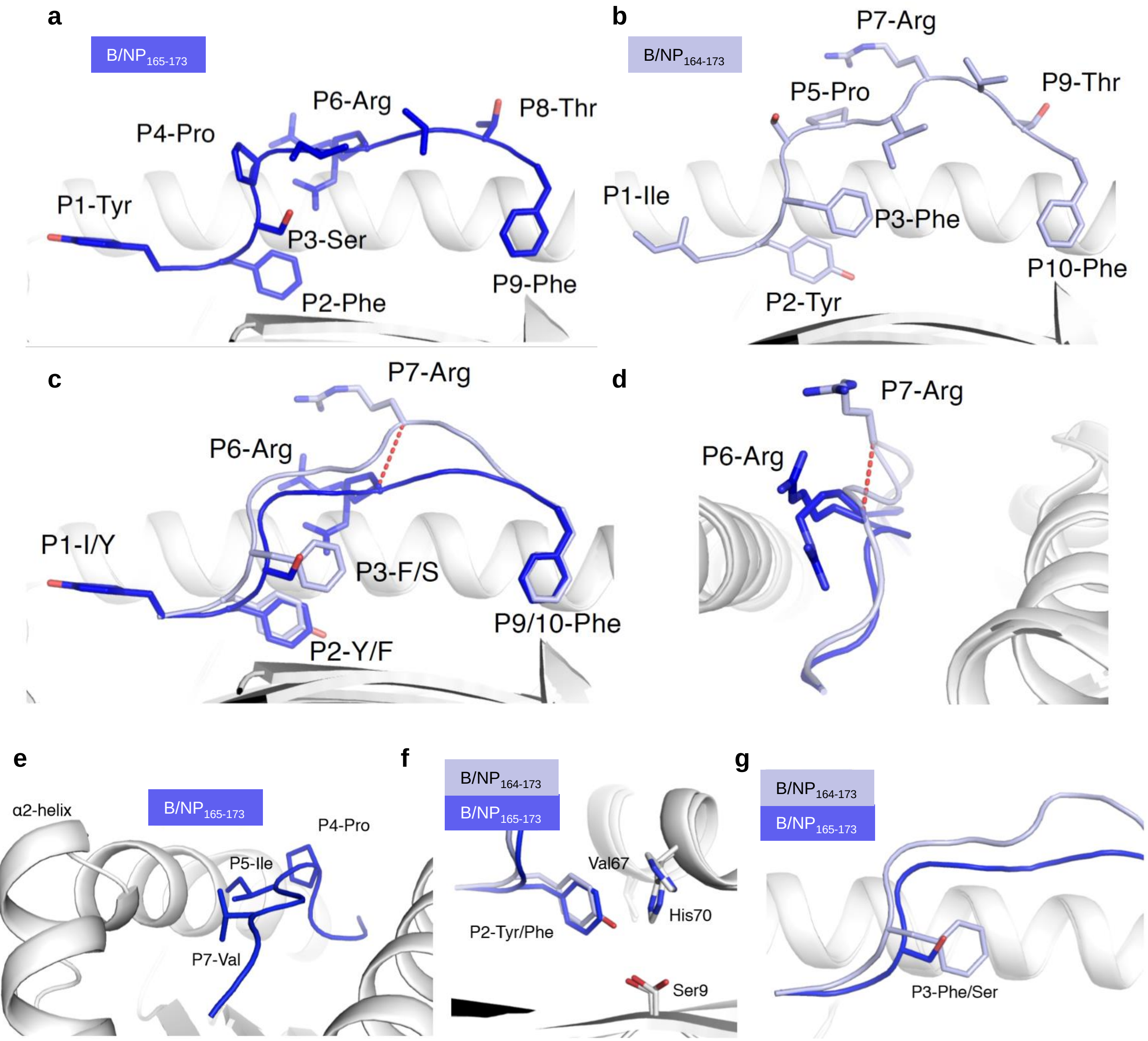

Supplementary Figure 7. Crystal structures of HLA-A*24:02 presenting B/NP$_{164-173}$ and B/NP$_{165-173}$ peptides. (a-c) Structures of B/NP$_{165-173}$ (**a**, blue stick), B/NP$_{164-173}$ (**b**, light blue stick) and **(c)** an overlay of B/NP$_{165-173}$ (blue) and B/NP$_{164-173}$ (light blue) presented on the HLA-A*24:02 molecule. **(d)** N-terminal side view overlay of B/NP$_{165-173}$ (blue) and B/NP$_{164-173}$ (light blue) peptides showing the largest structural difference at the Cα atom of the P6/7-Arg shown by the red dashed line between B/NP$_{165-173}$ (blue) and B/NP$_{164-173}$ (light blue), respectively. **(e)** The P5-Ile and P7-Val of the B/NP$_{165-173}$ 9mer peptide (blue) are half-buried between the peptide backbone and the HLA α2-helix, and together with the P4-Pro, form a hydrophobic patch at the centre of the peptide. **(f)** The substitution of P2-Tyr (B/NP$_{164-173}$) (light blue) for P2-Phe (B/NP$_{165-173}$) (blue) occurs without major structural rearrangement of the B pocket residues (white sticks), as both residues are large and aromatic. **(g)** The additional residue of B/NP$_{164-173}$ (light blue) changes the secondary anchor residue at P3 of the peptides from a small P3-Ser (B/NP$_{165-173}$) to a large P3-Phe (B/NP$_{164-173}$).

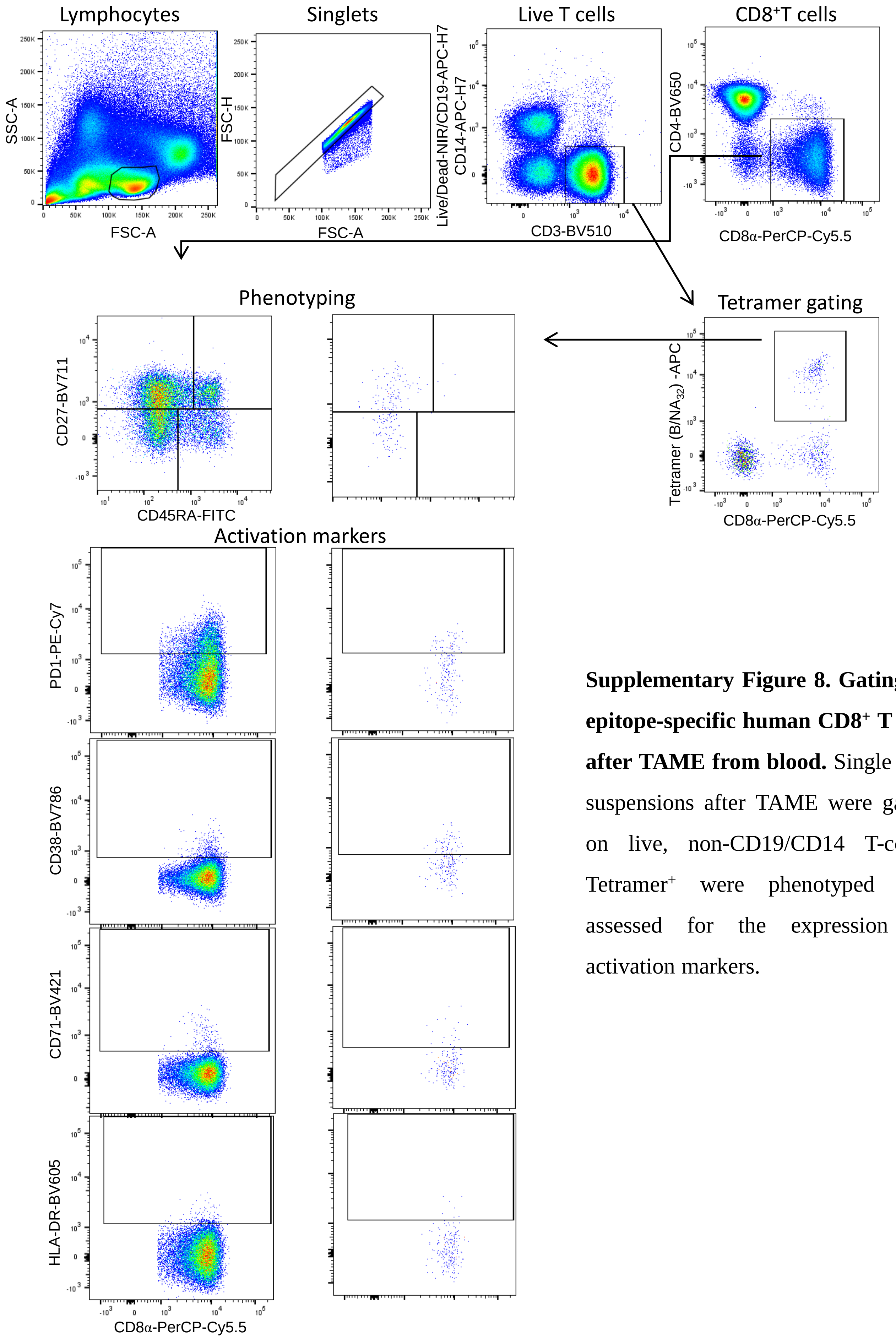

Supplementary Figure 8. Gating of epitope-specific human CD8+ T cell after TAME from blood. Single cell suspensions after TAME were gated on live, non-CD19/CD14 T-cells. Tetramer+ were phenotyped and assessed for the expression of activation markers.

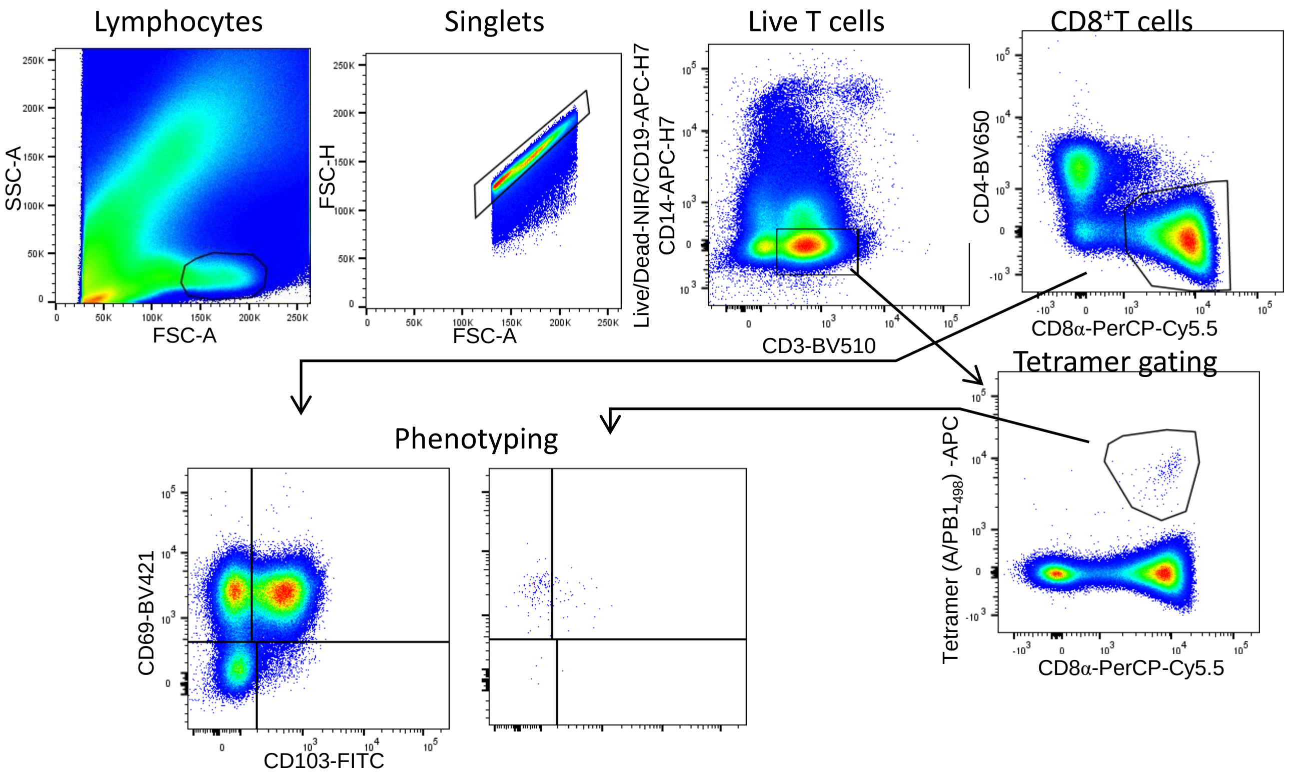

Supplementary Figure 9. Gating of epitope-specific human CD8+ T cell after TAME from tissues. Single cell suspensions after TAME were gated on live, non-CD19/CD14 T-cells. Tetramer+ were phenotyped.

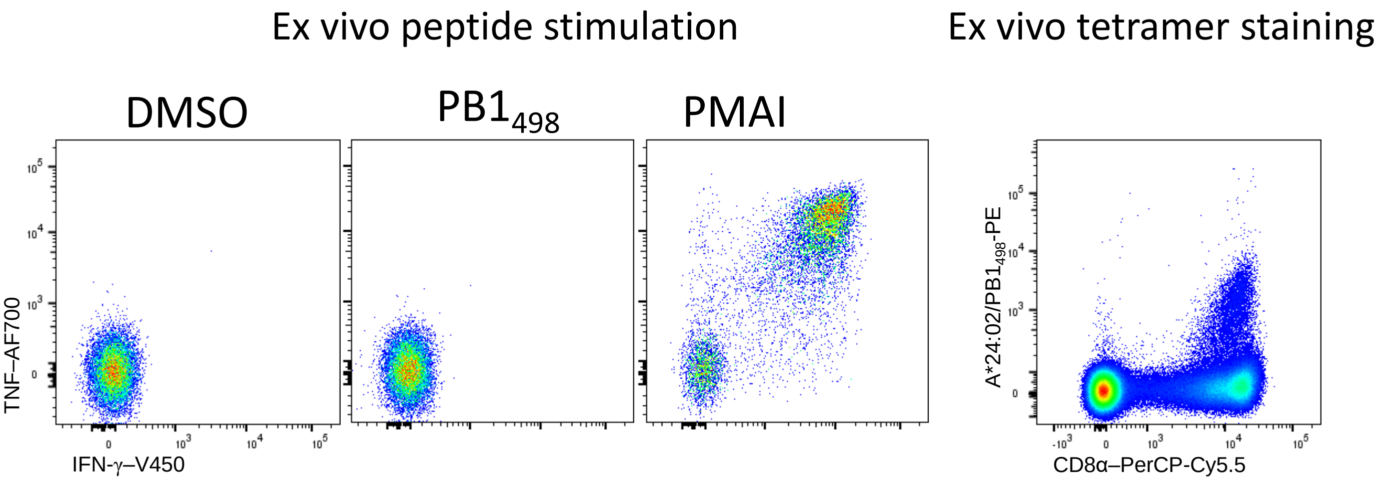

Supplementary Figure 10. Response to *ex vivo* A/PB1$_{498\text{-}505}$ peptide stimulation in unconventional Tetramer binding donor. PBMCs from non-LIFT8 were stimulated with A/PB1$_{498\text{-}505}$ peptide, DMSO (neg. control) or PMA+Ionomycin (pos. control) for a 6 h ICS to assess if unconventional *ex vivo* binding Tetramer^{+}CD8^{+} T cells are peptide reactive.

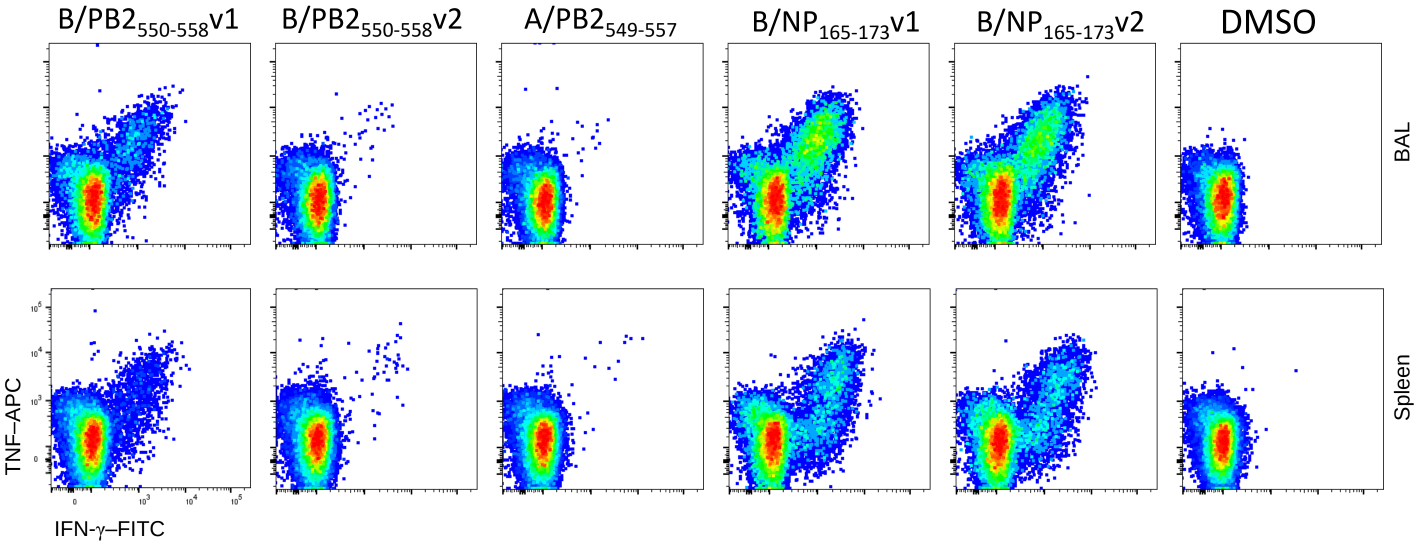

Supplementary Figure 11. Cross-reactive of CD8$^+$T cells in HHD-A24 after primary B/Malaysia infection. HHD-A24 were infected with B/Malaysia/2506/04 and 10 days after infection single cell suspensions stimulated in an ICS with the B/Malaysia/2506/04 derived peptide variant (V1) or with other naturally occurring variants derived by IBV or IAV.

Supplementary Data 1. Peptides identified from IAV (HKx31) and IBV (B/Malaysia)

Sequences of IAV (HKx31) and IBV (B/Malaysia)-derived peptides identified by LC-MS/MS analysis of the HLA class I and II immunopeptidomes of CIR and CIR.A24. For each peptide, the modifications with which it was identified, source protein, start site within the source protein, and the confidence of assignment for the data sets within which it was identified, are noted. For each data set, the cell line, infecting virus, antibody used (and any antibody depletion prior), time of infection and confidence cut-off for a 5% FDR are shown. Identifications above this confidence in each data set are in bold, those below are in italics and have increased likelihood of being false positive identifications. Naming of data sets match those in Supplementary Fig. 1. The predicted binding affinities (nM) and %rank for HLA-A*24:02, HLA-B*35:03 and HLA-C*04:01 for all 8-14mer peptides as calculated by NetMHCpan4.0 are shown. For HKx31, data sets derived from the sequential isolation of HLA from the same sample are noted. For B/Malaysia, previous identification in HLA isolations from B/Malaysia infected CIR and CIR.A*02:01 in Koutsakos *et al.*[24] are also noted. Colour fill represents isolations with w632 (blue), DT9 (yellow) and mixed class II antibodies (green). The "Best Explanation" column denotes the HLA hypothesised to present a given peptide based on appearance across the data sets and predicted binding by NetMHCpan4.0.

Supplementary Table 1. Donor demographics

Donor ID	Age group [years]	Sample	HLA-A	HLA-B	HLA-C	HLA-DRB1	HLA-DPB1	HLA-DQB1	IAV peptide expansion	IBV peptide expansion	IBV virus expansion	IAV virus expansion	TAME
LIFT1	40-60	PBMC	24:02, 34:01	15:21, 56:01	01:02, 04:03	04:12, 14:08	05:01, 16:01	04:02, 05:03	X				
LIFT2	40-60	PBMC	24:02	13:01, 56:01	01:02, 04:01	08:03, 14:02	04:01, 05:01	03:01, 06:01		X			
LIFT3	60-80	PBMC	24:02	13:01, 40:02	01:02, 04:01	08:03	02:01, 04:01	06:01	X				
LIFT4	60-80	PBMC	11:01, 24:02	39:01, 40:10	04:03, 12:03	08:03	02:01, 05:01	06:01	X				
LIFT5	18-40	PBMC	02:01, 24:02	15:21, 56:01	04:03, 07:02	04:05, 14:08	05:01, 14:01	04:01, 05:03		X			
LIFT6	18-40	PBMC	02:01, 24:02	07:02, 40:06	03:04, 07:02	11:01, 15:01	05:01	03:01, 06:02	X				
LIFT7	40-60	PBMC	01:01, 24:02	08:01, 15:21	04:03, 07:01	03:01, 14:07	14:01, 16:01	02:01, 05:03		X			
LIFT8	18-40	PBMC	24:02	40:01, 56:02	01:02, 03:03	08:03, 15:02	05:01	06:01		X			
LIFT9	18-40	PBMC	24:02, 34:01	07:02, 56:01	01:02, 07:02	04:12, 15:01	02:01, 04:01	04:02, 06:02		X			
LIFT10	18-40	PBMC	24:02	40:01, 40:02	01:02, 03:03	08:03, 15:02	02:01, 05:01	06:01	X				
LIFT11	18-40	PBMC	24:02, 34:01	13:01	04:01, 15:02	04:12, 12:01	05:01	03:01, 04:02		X			
LIFT12	18-40	PBMC	11:01, 24:02	13:01, 40:02	04:01, 15:02	04:05, 08:03	02:01, 04:01	03:01, 06:01		X			
LIFT13	18-40	PBMC	24:02	13:01, 56:01	01:02,03:03	14:08	04:01	05:03		X			
LIFT14	18-40	PBMC	24:02, 24:03	40:01, 52:01	03:03, 12:02	04:12, 15:02	04:01, 05:01	04:02, 06:01					X
LIFT15	18-40	PBMC	24:02	40:01, 56:02	01:02, 03:03	08:03	02:01	05:03, 06:01					X
LIFT16	18-40	PBMC	01:01, 24:02	56:01, 57:01	01:02, 06:02	07:01,15:02	04:01, 05:01	03:01, 03:02					X
LIFT17	18-40	PBMC	24:02, 24:06	40:01, 56:01	01:02, 03:03	04:12, 14:34	05:01	04:02, 05:03					X
Non-LIFT1	18-40	PBMC	01:01, 24:02	08:01, 14:02	n.D.	n.D.	n.D.	n.D.	X				
Non-LIFT2	18-40	PBMC	03:01, 24:02	35:03, 44:02	n.D.	n.D.	n.D.	n.D.	X				
Non-LIFT3	n.D.	PBMC	02:07, 24:02	46:01, 58:01	n.D.	n.D.	n.D.	n.D.	X				
Non-LIFT4	n.D.	PBMC	03:01, 24:02	15:01, 47:01	n.D.	n.D.	n.D.	n.D.	X				
Non-LIFT5	n.D.	PBMC	24:02	40:02, 51:01	n.D.	n.D.	n.D.	n.D.	X				
Non-LIFT6	40-60	PBMC	03:01, 24:02	15:01, 38:01	03:03, 12:03	13:01	n.D.	n.D		X	X	X	
Non-LIFT7	40-60	PBMC	02:01, 24:02	15:01, 35:02	01:02, 04:01	08:01, 11:04	n.D.	n.D.		X	X	X	
Non-LIFT8	40-60	PBMC	24:02	07:02	07:02	01:01, 10:01	n.D.	n.D		X	X	X	X
Non-LIFT9	18-40	PBMC	01:01, 24:02	35:01, 57:01	04:01, 06:02	01:01, 14:54	n.D.	n.D		X	X	X	
Non-LIFT10	18-40	PBMC	02:07, 24:02	46:01, 67:01	01:02, 07:02	08:03, 09:01	n.D.	n.D		X	X	X	X
Non-LIFT11	40-60	PBMC	24:02, 25:01	07:02, 18:01	n.D.	n.D.	n.D.	n.D.	X				X
Non-LIFT12	18-40	PBMC	02:03, 24:02	27:06, 40:01	03:04, 04:01	n.D.	n.D.	n.D.					X
Non-LIFT13	18-40	PBMC	01:01, 24:02	08:01, 40:01	03:04, 07:01	04:04/23, 11:01	n.D.	n.D.					X
Non-LIFT 14	18-40	PBMC	01:01, 24:02	44:02, 57:01	06:02, 16:04	04:02, 07:01	n.D.	n.D.					X
IAV-Inf 1	40-60	PBMC	01:01, 24:02	08:01, 57:01	06:02, 07:01	03:01, 07:01	01:01, 04:01	02:01, 03:03					X
IAV-Inf 2	80-99	PBMC	24:02, 29:02	18:01, 51:01	07:01, 16:02	04:03, 15:01	01:01, 04:01	03:02, 06:02					X
IAV-Inf 3	18-40	PBMC	24:02, 32:01	07:02, 56:01	01:02, 07:02	01:01	04:01; 06:01	05:01					X
IAV-Inf 4	60-80	PBMC	24:02, 74:01	07:02, 51:01	01:02, 15:02	04:03, 15:01	04:01	03:02, 06:02					X
IBV-Inf 1	40-60	PBMC	11:01, 24:02	08:01, 15:01	03:03, 07:01	04:01, 15:01	03:01	04:02					X
IBV-Inf 2	60-80	PBMC	24:02, 24:03	40:06, 51:06	12:04, 15:07	04:03, 07:01	01:01, 02:01	03:02, 03:03					X
IBV-Inf 3	60-80	PBMC	03:01, 24:02	07:02, 51:01	0x:03, 07:02	09:01, 15:01	n.D.	03:03, 06:02					X
Sp1	40-60	Spleen	02:01, 24:02	27:05, 35:01	02:01, 04:01	01:01, 12:01	n.D.	n.D.					X
Sp2	40-60	Spleen	02:01, 24:02	07:02, 15:01	03:01, 07:01	13:01, 15:01	n.D.	n.D.					X
Sp3	40-60	Spleen	02:01, 24:02	27:05, 35:01	02:01, 04:01	01:01, 11:01	n.D.	n.D.					X
Sp4	60-80	Spleen	02:01, 24:02	07:02, 15:01	03:01, 07:01	13:01, 15:01	n.D.	n.D.					X
LN1	40-60	Pancreatic Lymphnode	02:01, 24:02	27:05, 35:01	02:01, 04:01	01:01, 12:01	n.D.	n.D.					X
TN1	0-10	Tonsil	24:02, 29:02	27:05, 42:01	02:02, 17:01	04;01, 08:04	02:01	03:01, 03:02					X
TN2	40-60	Tonsil	24:02, 32:01	15:01, 44:02	03:03, 05:01	04:01, 15:01	04:01, 05:01	03:02, 06:02					X
TN3	0-10	Tonsil	24:02, 29:02	51:01, 58:01	03:03, 07:18	08:04, 15:01	04:01	04:02, 06:02					X
L1	40-60	Lung	3, 24	15, 55	3, 3	n.D	n.D	n.D					X

n.D. = not determined

Supplementary Table 2. Influenza A peptides identified by immunopeptidomics.

Peptide	Sequence	Virus origin	Source	Peptide Pool	Conservation[1]	Predicted binding affinity (nM)[2]	Comb. CTL epitope score[3]	Immunogenic in human donors (of tested)	Immunogenic in mice (of tested)	Immunogenic & presented by other HLA/MHC[4]
PA$_{47-53}$	MYSDFHF	IAV	MS	5	1				0/10	-
PA$_{47-60}$	MYSDFHFINEQGES	IAV	MS	5	0.52	18148			0/10	-
PA$_{48-53}$	YSDFHF	IAV	MS	5	1				0/10	-
PA$_{130-138}$	YYLEKANKI	IAV	MS/iedb	1	1	29		1/10	1/10	A*23:01
PA$_{457-465}$	EYIMKGVYI	IAV	MS	3	0.99	590	0.50		1/10	-
**PA$_{649-658}$**	LYASPQLEGF	IAV	MS/iedb	1	0.99	59	0.81	5/10	0/10	A*11:01
PB1$_{2-10}$	DVNPTLLFL	IAV	MS	5	0.98	22015	0.29		4/10	-
PB1$_{19-26}$	STTFPYTG	IAV	MS	5	1	40250	0.09		0/10	-
PB1$_{216-222}$	SYLIRAL	IAV	MS	5	0.19				0/10	-
PB1$_{216-224}$	SYLIRALTL	IAV	MS	1	0.19	40	0.89	2/5	10/10	-
PB1$_{430-438}$	RYTKTTYWW	IAV	MS	1	0.15	26	0.98	2/5	0/10	-
PB1$_{482-490}$	SYINRTGTF	IAV	MS	1	0.16	23	1.03	0/5	0/10	-
PB1$_{482-492}$	SYINRTGTFEF	IAV	MS	1	0.14	20	1.07	0/5	0/10	-
**PB1$_{496-505}$**	FYRYGFVANF	IAV	iedb	2	0.99	62	0.90	9/10	2/10	A*23:01, A*29:02
**PB1$_{498-505}$**	RYGFVANF	IAV	MS/iedb	1	0.99	40	1.05	9/10	10/10	-
PB1$_{500-505}$	GFVANF	IAV	MS	5	0.99				0/10	-
PB1-1$_{746-755}$	QYKGKLCL	IAV	MS	4		18075	0.53		0/10	-
PB1+2$_{8-16}$	EWMSIRPYF	IAV	MS	2		88	0.64	1/5	0/10	-
PB1+3$_{24-32}$	CYKHNFPLY	IAV	MS	3		730	0.73		0/10	-
PB1+3$_{682-689}$	QYGGGYGF	IAV	MS	3		1134	0.81		1/10	-
PB2$_{89-98}$	VMVSPLAVTW	IAV	MS	3	0.91	2179	0.52		0/10	-
PB2$_{110-119}$	HYPKIYKTYF	IAV	MS	1	0.11	28	0.89	3/5	0/10	-
PB2$_{112-119}$	PKIYKTYF	IAV	MS	5	0.12	39253	0.32		0/10	-
PB2$_{114-122}$	IYKTYFERV	IAV	MS	2	0.01	157	0.74	0/5	0/10	-
PB2$_{117-125}$	TYFERVERL	IAV	MS	1	0.01	57	0.69	0/10	0/5	-
PB2$_{204-212}$	AYMLERELV	IAV	MS	2	0.99	194	0.49	0/5	0/10	-
PB2$_{227-234}$	VYIEVLHL	IAV	MS	2	0.59	361	0.87	0/5	1/10	H2K^{b}
PB2$_{322-330}$	SFSFGGFTF	IAV	MS	1	0.99	20	0.95	0/5	10/10	-
PB2$_{437-446}$	HFQKDAKVLF	IAV	MS	3	0.96	392	0.67		1/10	-
PB2$_{463-471}$	ILPDMTPSI	IAV	MS	3	0.01	1606	0.51		10/10	-
PB2$_{549-555}$	TYQWIIR	IAV	MS	5	0.97				0/10	-
**PB2$_{549-557}$**	TYQWIIRNW	IAV	MS	2	0.96	62	0.86	4/8	10/10	-
PB2$_{549-559}$	TYQWIIRNWET	IAV	MS	4	0.17	8570	0.29		10/10	-
PB2$_{552-559}$	WIIRNWET	IAV	MS	5	0.17	36284	0.04		0/10	-
PB2$_{591-599}$	QYSGFVRTL	IAV	MS	2	0.44	214	0.61	1/5	1/10	-
PB2$_{591-600}$	QYSGFVRTLF	IAV	MS	1	0.44	20	0.85	0/5	0/5	-
PB2$_{594-600}$	GFVRTLF	IAV	MS	5	0.99				0/10	-
PB2$_{703-710}$	RYGPALSI	IAV	MS	2	0.98	389	0.88	1/5	4/10	-
HA$_{176-184}$	TYPVLNVTM	IAV	MS	2	0.01	351	0.70	1/5	0/10	-
HA$_{248-259}$	IYWTIVKPGDVL	IAV	MS	3	0.01	958			1/10	-
HA$_{506-516}$	VYRDEALNNRF	IAV	MS	3	0.40	556	0.68		0/10	-
HA$_{507-516}$	YRDEALNNRF	IAV	MS	5	0.41	20902	0.28		0/10	-
HA$_{508-516}$	RDEALNNRF	IAV	MS	5	0.41	28117	0.27		0/10	-
M1$_{99-109}$	LYRKLKREITF	IAV	iedb	3	0.48	409	0.70		0/10	-
M1$_{108-117}$	TFHGAKEISL	IAV	iedb	4	0.00	1274	0.39		0/10	-
M1$_{239-248}$	AYQKRMGVQM	IAV	iedb	4	0.66	3281			0/10	A*11:01
NP$_{39-47}$	FYIQMCTEL	IAV	iedb	2	0.99	61	0.63	1/10	6/10	H2-D^{b}, H2-D^{b}, A*23:01, A*29:02, H2-K^{d}
NP$_{138-148}$	IWHSNLNDATY	IAV	MS	4	0.72	10945	0.54		0/10	-
NP$_{257-265}$	TFLARSALI	IAV	MS	2	0.12	218	0.65	1/5	0/10	-
NP$_{296-304}$	YSLVGIDPF	IAV	MS	3	0.9	1317	0.60		0/10	H2-D^{b}
NP$_{417-425}$	NLPFDRTTI	IAV	MS	4	0.01	15720	0.34		2/10	-
NP$_{419-429}$	PFDRTTIMAAF	IAV	iedb	3	0.01	5016	0.51		0/10	-
NP$_{456-464}$	VSFQGRGVF	IAV	MS	4	0.14	7866	0.39		1/10	-
NS2$_{98-106}$	TFMQALHLL	IAV	MS	1	0.01	32	0.80	3/5	0/10	-

Boldface highlights immunodominant epitopes in human donors

Underline highlights immunogenic peptides in HHD-A24 mice

[1]Unique amino acid sequences of protein origin corresponding to the identified peptides from influenza A and B viruses were sourced from the NCBI database using full length sequences from human hosts isolated in Asia and/or Australia. Influenza A sequences were limited to H1N1 and H3N2 human isolate sequences. Sequence variants were identified using the *Identify short peptide in proteins* tool from the Influenza Research Database (www.fludb.org).

[2] Analysed with NetpanMHC 4.0 (http://www.cbs.dtu.dk/services/NetMHCpan-4.0/)

[3] Analysed with NetCTLpan 1.1 (http://www.cbs.dtu.dk/services/NetCTLpan/)

[4] Peptides were screened on the immune epitope database (www.iedb.org) to search for positive T cell assays.

Supplementary Table 3. Influenza B peptides identified by immunopeptidomics

Peptide	Sequence	Virus origin	Peptide pool	Conservation[1]	Predicted binding affinity (nM)[2]	Comb. CTL epitope score[3]	Immunogenic in human donors (of tested)	Immunogenic in mice (of tested)	Immunogenic & presented by other HLA/MHC[4]
$PA_{146-156}$	MIFSYNQDYSL	IBV	11	0.99	2146	0.38		2/10	-
$PA_{453-461}$	TVMMKYVLF	IBV	11	0.99	122	0.58		0/14	-
**$PA_{457-465}$**	KYVLFHTSL	IBV	10	0.96	220	0.72	8/14	0/14	-
$PB1_{495-504}$	FYRDGFVSNF	IBV	10	0.99	312	0.90	2/14	0/14	-
**$PB1_{503-511}$**	NFAMELPSF	IBV	10	0.39	473	0.58	7/14	0/14	-
$PB2_{103-113}$	TYGPIGDTEGF	IBV	10	0.93	357	0.84	3/14	0/14	-
$PB2_{245-253}$	IYHPGGNKL	IBV	11	0.99	384	0.70		3/14	-
$PB2_{405-413}$	VFSQDTRMF	IBV	11	0.99	343	0.60		0/14	-
$PB2_{433-441}$	MYQLQRYFL	IBV	10	1	219	0.84	6/14	0/14	-
$PB2_{439-448}$	YFLNRSNDLF	IBV	10	0.65	63	0.87	5/14	0/14	-
**$PB2_{550-558}$**	TYQWVLKNL	IBV	10	0.85	335	0.80	11/14	9/14	-
$HA_{219-228}$	LYGDSKPQKF	IBV	10	0.34	111	0.82	4/14	0/14	-
$HA_{238-246}$	HYVSQIGGF	IBV	11	0.68	728	0.62		0/14	-
$HA_{338-346}$	IWVKTPLKL	IBV	10	0.98	247	0.79	1/14	3/14	-
$HA_{339-346}$	WVKTPLKL	IBV	11	0.98	15986	0.30		1/10	-
$HA_{370-380}$	GFLEGGWEGMI	IBV	11	0.99	18807	0.44		1/9	-
**$HA_{552-560}$**	YYSTAASSL	IBV	10	0.99	73	0.68	9/14	0/14	$H2\text{-}K^d$
$HA_{571-579}$	VYMVSRDNV	IBV	11	0.89	2295	0.37		0/14	$H2\text{-}K^d$
$HA+3_{276-284}$	NYLSKRYFI	IBV	10	n.d.	73	0.75	4/14	0/14	-
$M1_{131-140}$	MYLNPGNYSM	IBV	10	0.80	642	0.72	7/14	0/14	-
NA_{1-12}	MLPSTIQTLTLF	IBV	11	0.98	3376	n.d.		1/14	-
NA_{32-39}	LYSDILLK	IBV	11	0.91	24963	0.35		0/10	-
**NA_{32-40}**	LYSDILLKF	IBV	10	0.91	44	1.04	9/14	14/14	-
$NA_{209-217}$	AYTDTYHSY	IBV	11	0.99	4132	0.62		0/13	-
$NA_{213-221}$	TYHSYANNI	IBV	10	0.25	65	0.69	6/14	11/14	-
$NA_{402-411}$	SMEEPGWYSF	IBV	11	0.63	943	0.60		1/14	-
$NA_{458-466}$	TVTGVNMAL	IBV	11	0.10	23313	0.31		0/10	-
$NA+3_{480-489}$	PFVPILFEQL	IBV	11		2296	0.59		0/14	-
NP_{79-89}	VYNMVVKLGEF	IBV	11	1	954	0.77		0/14	-
**$NP_{164-173}$**	IYFSPIRVTF	IBV	10	0.17	25	1.00	11/14	14/14	-
**$NP_{165-173}$**	YFSPIRVTF	IBV	10	0.17	65	1.05	12/14	14/14	-
$NP_{181-191}$	MYKTTMGSDGF	IBV	11	0.97	2637	0.68		0/12	-
$NP_{218-228}$	VGLDPSLISTF	IBV	11	1	8661	0.51		0/10	-
$NP_{338-345}$	IYAKIPQL	IBV	11	1	1015	0.90		0/12	-
$NP_{338-347}$	IYAKIPQLGF	IBV	10	1	9	1.01	7/14	4/14	-
$NP_{392-400}$	AAYEDLRVL	IBV	11	0.99	36058	0.29		13/14	-
$NP_{399-408}$	VLSALTGTEF	IBV	11	0.97	3729	0.45		0/12	-
$NS1_{78-90}$	KAIGVKMMKVLLF	IBV	11	0.95	6046	n.d.		0/12	-
$NS1_{211-218}$	AYDQSGRL	IBV	11	0.89	25406	0.38		0/10	-
$NS1_{211-219}$	AYDQSGRLV	IBV	11	0.89	17951	0.29		0/10	-
$NS2_{28-37}$	VLMKDIQSQF	IBV	11	0.89	1134	0.62		0/12	-

Boldface highlights immunodominant epitopes in human donors

Underline highlights immunogenic peptides in HHD-A24 mice

[1]Unique amino acid sequences of protein origins corresponding to the identified peptides from influenza A and B viruses were sourced from the NCBI database using full length sequences from human hosts isolated in Asia and/or Australia. Influenza A sequences were limited to H1N1 and H3N2 human isolate sequences. Sequence variants were identified using the *Identify short peptide in proteins* tool from the Influenza Research Database (www.fludb.org).

[2] Analysed with NetpanMHC 4.0 (http://www.cbs.dtu.dk/services/NetMHCpan-4.0/)

[3] Analysed with NetCTLpan 1.1 (http://www.cbs.dtu.dk/services/NetCTLpan/)

[4] Peptides were screened on the immune epitope database (www.iedb.org) to search for positive T cell assays.

Supplementary Table 4. Screened IAV and IBV peptide variants

Peptide	Sequence	Origin	Conservation[1]
PB1$_{216-224}$v1	SYLIRALTL	IAV	0.19
PB1$_{216-224}$v2	GYLIRALTL	IAV	0.79
PB1$_{430-438}$v1	RYTKTTYWW	IAV	0.15
PB1$_{430-438}$v2	KYTKTIYWW	IAV	0.28
PB1$_{430-438}$v3	KYTKTTYWW	IAV	0.55
PB1$_{482-490}$v1	SYINRTGTF	IAV	0.16
PB1$_{482-490}$v2	SYINKTGTF	IAV	0.83
PB1$_{482-492}$v1	SYINRTGTFEF	IAV	0.14
PB1$_{482-492}$v2	SYINKTGTFEF	IAV	0.83
PB2$_{110-119}$v1	HYPKIYKTYF	IAV	0.11
PB2$_{110-119}$v1	HYPKVYKTYF	IAV	0.87
PB2$_{114-122}$v1	IYKTYFERV	IAV	0.01
PB2$_{114-122}$v2	VYKTYFEKV	IAV	0.47
PB2$_{114-122}$v3	IYKTYFEKV	IAV	0.11
PB2$_{114-122}$v4	VYKTYFDKV	IAV	0.40
PB2$_{117-125}$v1	TYFERVERL	IAV	0.01
PB2$_{117-125}$v2	TYFEKVERL	IAV	0.58
PB2$_{117-125}$v3	TYFDKVERL	IAV	0.40
PB2$_{227-234}$v1	VYIEVLHL	IAV	0.59
PB2$_{227-234}$v2	IYIEVLHL	IAV	0.39
PB2$_{227-234}$v3	MYIEVLHL	IAV	0.01
PB2$_{463-471}$v1	ILPDMTPSI	IAV	0.01
PB2$_{463-471}$v2	ILPDMTPST	IAV	0.57
PB2$_{463-471}$v3	VLPDMTPST	IAV	0.40
PB2$_{591-599}$v1	QYSGFVRTL	IAV	0.44
PB2$_{591-599}$v2	RYSGFVRTL	IAV	0.46
PB2$_{591-600}$v1	QYSGFVRTLF	IAV	0.44
PB2$_{591-600}$v2	RYSGFVRTLF	IAV	0.46
M1$_{99-109}$v1	LYRKLKREITF	IAV	0.48
M1$_{99-109}$v2	LYKKLKREITF	IAV	0.49
HA$_{176-184}$v1	TYPVLNVTM	IAV	0.01
HA$_{176-184}$v2	KYPALNVTM	IAV	0.34
HA$_{176-184}$v3	TYPALNVTV	IAV	0.05
HA$_{176-184}$v4	KYPVLNVTM	IAV	0.01
NP$_{257-265}$v1	TFLARSALI	IAV	0.12
NP$_{257-265}$v2	IFLARSALI	IAV	0.80
NP$_{257-265}$v3	IFSARSALI	IAV	0.07
NS2$_{98-106}$v1	TFMQALHLL	IAV	0.01
NS2$_{98-106}$v2	TFMQALQLL	IAV	0.96
NP$_{164-173}$v1	IYFSPIRVTF	IBV	0.17
NP$_{164-173}$v2	IYFSPIRITF	IBV	0.79
NP$_{165-173}$v1	YFSPIRVTF	IBV	0.17
NP$_{165-173}$v2	YFSPIRITF	IBV	0.79
NA$_{32-40}$v1	LYSDILLKF	IBV	0.91
NA$_{32-40}$v2	LYSDVLLKF	IBV	0.01
NA$_{213-221}$v1	TYHSYANNI	IBV	0.25
NA$_{213-221}$v2	TYHSYAKNI	IBV	0.41
NA$_{213-221}$v3	THYSYANKI	IBV	0.25
NA$_{213-221}$v4	THYSYAHNI	IBV	0.01
PB2$_{550-558}$v1	TYQWVLKNL	IBV	0.85
PB2$_{550-558}$v2	TYQWVMKNL	IBV	0.14
PB2$_{439-448}$v1	YFLNRSNDLF	IBV	0.65
PB2$_{439-448}$v2	YFLSRSNDLF	IBV	0.34
PB1$_{503-511}$v1	NFAMELPSF	IBV	0.39
PB1$_{503-511}$v2	NFAMEIPSF	IBV	0.61
M1$_{131-140}$v1	MYLNPGNYSM	IBV	0.80
M1$_{131-140}$v2	MYLNRGNYSM	IBV	0.02
M1$_{131-140}$v3	MYLNPENYSM	IBV	0.02
HA$_{219-228}$v1	LYGDSKPQKF	IBV	0.34
HA$_{219-228}$v2	LYGDSNPQKF	IBV	0.61

[1]Unique amino acid sequences of protein origins of the identified peptides of influenza A and B viruses were sourced from the NCBI database using full length sequences from human hosts isolated in Asia and/or Australia. Influenza A sequences were limited to H1N1 and H3N2 human isolate sequences. Sequence variants were identified using the *Identify short peptide in proteins* tool from the Influenza Research Database (www.fludb.org).

Supplementary Table 5. Data Collection, Refinement Statistics and Thermal stability of peptide-HLA complexes

**Data Collection Statistics**	**HLA-A*24:02-PB2$_{549-559}$**	**HLA-A*24:02-PB2$_{549-557}$**	**HLA-A*24:02-PB2$_{549-557B}$**	**HLA-A*24:02-NP$_{165-173}$**	**HLA-A*24:02-NP$_{164-173}$**
Space group	**I2**	**P6$_5$22**	**P2$_1$**	**P2$_1$2$_1$2$_1$**	**C222$_1$**
Cell Dimensions (a,b,c) (Å)	89.68, 43.65, 236.49, β=95.56°	87.29, 87.29, 312.46	46.07, 123, 86.59 β=103.4°	62.95, 75.55, 89.91	79.92, 120.25, 187.80
Resolution (Å)	39.23 – 2.95 (3.13 – 2.95)	48.16 – 2.90 (3.08 – 2.90)	44.81 – 2.16 (2.23 – 2.16)	34.91 – 1.51 (1.54 – 1.51)	46.95 – 2.75 (2.90 – 2.75)
Total number of observations	73078 (11941)	159459 (25130)	188348 (15744)	625371 (30507)	158168 (24193)
Number of unique observations	19624 (3149)	16623 (2577)	49524 (4188)	64117 (3233)	23763 (3421)
Multiplicity	3.7 (3.8)	9.6 (9.8)	3.8 (3.8)	9.8 (9.4)	6.7 (7.1)
Data completeness (%)	99.6 (99.4)	99.9 (99.9)	99.2 (96.9)	94.0 (96.2)	99.4 (99.3)
I/σ_I	12.3 (2.0)	16.4 (2.1)	14.4 (2.6)	28.4 (4.4)	7.8 (1.9)
R_{pim}[a] (%)	6.7 (40.4)	2.9 (31.1)	4.6 (31.9)	1.4 (14.9)	7.5 (40.3)
**Refinement Statistics**					
Non-hydrogen atoms	6405	3177	6898	3914	6387
Protein	6328	3159	6309	3320	6295
Water	77	14	587	594	91
R_{factor}[b] (%)	20.3	24.1	17.8	18.2	18.9
R_{free}[b] (%)	27.6	27.4	22.9	20.6	26.1
Rms deviations from ideality					
Bond lengths (Å)	0.010	0.007	0.010	0.006	0.010
Bond angles (°)	1.16	0.96	1.08	0.87	1.16
Ramachandran plot (%)					
Favoured region	91.0	93.0	99.3	98.0	94.0
Allowed region	8.0	6.0	0.7	2.0	5.0
Disallowed region	1.0	1.0	0.0	0.0	0.0
Average B-factor					
Macromolecules	63.81	108.46	34.63	21.60	56.82
Water molecules	36.60	91.60	40.54	34.62	45.69
Ligand	NA	115.80	39.06	30.85	78.83
**Tm (C)**	57.1 ± 1.3	61.9 ± 1.3	57.1 ± 1.2	57.5 ± 0.3	64.0 ± 2.2

[a] $R_{p.i.m} = \Sigma_{hkl} [1/(N-1)]^{1/2} \Sigma_i | I_{hkl, i} - \langle I_{hkl} \rangle | / \Sigma_{hkl} \langle I_{hkl} \rangle$, [b] $R_{factor} = \Sigma_{hkl} | | F_o | - | F_c | | / \Sigma_{hkl} | F_o |$ for all data except ≈ 5% which were used for R_{free} calculation.

Supplementary Table 6. Staining panels

Panel 1 (Mouse ICS)			
**Antibody**	**Flourochrome**	**Vendor (clone/cat. No)**	**Dilution**
Live/Dead	NIR	Invitrogen (L34976)	1:800
CD8	PerCP-Cy 5.5	BD Pharmingen (53-6.7/551162)	1:350
CD4	PE-Cy7	eBioscience (GK1.5/25-0041-82)	1:200
IL-2	PE	BD Pharmingen (JES6-5H4/554428)	1:200
TNF	APC	BD Pharmingen (MP6-XT22/554420)	1:200
IFNγ	FITC	BioLegend (XMG1.2/505806)	1:200

Panel 2 (Human ICS)			
**Antibody**	**Flourochrome**	**Vendor (clone/cat. No)**	**Dilution**
Live/Dead	NIR	Invitrogen (L34976)	1:800
CD3	PE-Cy7	BD Pharmingen (UCHT1/563423)	1:50
CD4	PE	BD Pharmingen (RPA-T4/555347)	1:50
CD8	PerCP-Cy5.5	BD Pharmingen (SK1/565310)	1:100
CD107a	AF488	Invitrogen (eBioH4A3/53107942)	1:200
IFNγ	BV421	BD Horizon (B27/560371)	1:100
TNF	AF700	BD Pharmingen (MAb11/557996)	1:50
MIP-1β	APC	BD Pharmingen (D21-1351/560686)	1:40

Panel 3 (PBMC TAME)			
**Antibody**	**Flourochrome**	**Vendor (clone/cat. No)**	**Dilution**
CD71	BV421	BD Horizon (M-A712/562995)	1:50
CD3	BV510	BioLegend (OKT3/317332)	1:200
HLA-DR	BV605	BioLegend (L243/307640)	1:100
CD4	BV650	BD Horizon (SK3/563875)	1:200
CD27	BV711	BD Horizon (L128/563167)	1:200
CD38	BV786	BD Horizon (HIT2/563964)	1:100
Tetramer 1	APC	In house	
CCR7	AF700	BD Pharmingen (150503/561143)	1:50
CD14	APC-H7	BD Pharmingen (MφP9/560180)	1:100
CD19	APC-H7	BD Pharmingen (SJ25C1/560177)	1:100
Live/Dead	NIR	Invitrogen (L34976)	1:800
CD45RA	FITC	BD Pharmingen (HI100/555488)	1:200
CD8	PerCp-Cy5.5	BD Pharmingen (SK1/565310)	1:50
Tetramer 2	PE	In house	
CD95	PE-CF594	BD Horizon (DX2/562395)	1:100
PD1	PE-Cy7	BD Pharmingen (EH12.1/561272)	1:50

Panel 4 (Tissue TAME)			
**Antibody**	**Flourochrome**	**Vendor (cat. No)**	**Dilution**
CD69	BV421	BioLegend (FN50/310930)	1:100
CD3	BV510	BioLegend (OKT3/317332)	1:200
HLA-DR	BV605	BioLegend (L243/307640)	1:100
CD4	BV650	BD Horizon (SK3/563875)	1:200
CD27	BV711	BD Horizon (L128/563167)	1:200
CD38	BV786	BD Horizon (HIT2/563964)	1:100
Tetramer 1	APC	In house	
CCR7	AF700	BD Pharmingen (150503/561143)	1:50
CD14	APC-Cy7	BD Pharmingen (MφP9/560180)	1:100
CD19	APC-Cy7	BD Pharmingen (SJ25C1/560177)	1:100
Live/Dead	NIR	Invitrogen (L34976)	1:800
CD103	FITC	BioLegend (Ber-ACT8/350203)	1:100
CD8	PerCp-Cy5.5	BD Pharmingen (SK1/565310)	1:50
Tetramer 2	PE	In house	
CD95	PE-CF594	BD Horizon (DX2/562395)	1:100
CD45RO	PE-Cy7	ThermoFisher (UCHL1/25-0457-41)	1:100

Supplementary Table 7. Primers

Primer	Sequence (5’ – 3’)
Mycopl-for	YGCCTGVGTAGTAYRYWCGC
Mycopl-rev	GCGGTGTGTACAARMCCCGA
