# Supplementary material for: CD8+ T cell landscape in Indigenous and non-Indigenous people restricted by influenza mortality-associated HLA-A*24:02 allomorph
Source: Nat Commun. 2021 May 18;12:2931. doi: 10.1038/s41467-021-23212-x (PMC8132304; doi:10.1038/s41467-021-23212-x)
Supplement: Supplementary file 3 — Descriptions of Additional Supplementary Files [file 41467_2021_23212_MOESM3_ESM.pdf]

Description of Additional Supplementary Files

Supplementary Data 1
Description: Peptides identified from IAV (HKx31) and IBV (B/Malaysia) Sequences of IAV (HKx31) and IBV (B/Malaysia)-derived peptides identified by LC-MS/MS analysis of the HLA class I and II immunopeptidomes of CIR and CIR.A24. For each peptide, the modifications with which it was identified, source protein, start site within the source protein, and the confidence of assignment for the data sets within which it was identified, are noted. For each data set, the cell line, infecting virus, antibody used (and any antibody depletion prior), time of infection and confidence cut-off for a 5% FDR are shown. Identifications above this confidence in each data set are in bold, those below are in italics and have increased likelihood of being false positive identifications. Naming of data sets match those in Supplementary Fig. 1. The predicted binding affinities (nM) and %rank for HLA-A*24:02, HLAB*35:03 and HLA-C*04:01 for all 8-14mer peptides as calculated by NetMHCpan4.0 are shown. For HKx31, data sets derived from the sequential isolation of HLA from the same sample are noted. For B/Malaysia, previous identification in HLA isolations from B/Malaysia infected CIR and CIR.A*02:01 in Koutsakos *et al.*[24] is also noted. Colour fill represents isolations with w632 (blue), DT9 (yellow) and mixed class II antibodies (green). The “Best Explanation” column denotes the HLA hypothesised to present a given peptide based on appearance across the data sets and predicted binding by NetMHCpan4.0.
