# Supplementary material for: CD8+ T cell landscape in Indigenous and non-Indigenous people restricted by influenza mortality-associated HLA-A*24:02 allomorph
Source: Nat Commun. 2021 May 18;12:2931. doi: 10.1038/s41467-021-23212-x (PMC8132304; doi:10.1038/s41467-021-23212-x)
Supplement: Supplementary file 5 — Reporting Summary [file 41467_2021_23212_MOESM5_ESM.pdf]

nature research

Corresponding author(s): Katherine Kedzierska

Last updated by author(s): 15 Apr 2021

Reporting Summary

Nature Research wishes to improve the reproducibility of the work that we publish. This form provides structure for consistency and transparency in reporting. For further information on Nature Research policies, see our Editorial Policies and the Editorial Policy Checklist.

Statistics

For all statistical analyses, confirm that the following items are present in the figure legend, table legend, main text, or Methods section.

n/a	Confirmed	
☐	☒	The exact sample size (n) for each experimental group/condition, given as a discrete number and unit of measurement
☐	☒	A statement on whether measurements were taken from distinct samples or whether the same sample was measured repeatedly
☐	☒	The statistical test(s) used AND whether they are one- or two-sided<br>*Only common tests should be described solely by name; describe more complex techniques in the Methods section.*
☐	☒	A description of all covariates tested
☐	☒	A description of any assumptions or corrections, such as tests of normality and adjustment for multiple comparisons
☐	☒	A full description of the statistical parameters including central tendency (e.g. means) or other basic estimates (e.g. regression coefficient) AND variation (e.g. standard deviation) or associated estimates of uncertainty (e.g. confidence intervals)
☐	☒	For null hypothesis testing, the test statistic (e.g. F, t, r) with confidence intervals, effect sizes, degrees of freedom and P value noted<br>*Give P values as exact values whenever suitable.*
☒	☐	For Bayesian analysis, information on the choice of priors and Markov chain Monte Carlo settings
☒	☐	For hierarchical and complex designs, identification of the appropriate level for tests and full reporting of outcomes
☒	☐	Estimates of effect sizes (e.g. Cohen's d, Pearson's r), indicating how they were calculated

Our web collection on statistics for biologists contains articles on many of the points above.

Software and code

Policy information about availability of computer code

Data collection	BD FACS DIVA v8.0.1
Data analysis	FlowJo v10; Prism v8.3; R v4.0

For manuscripts utilizing custom algorithms or software that are central to the research but not yet described in published literature, software must be made available to editors and reviewers. We strongly encourage code deposition in a community repository (e.g. GitHub). See the Nature Research guidelines for submitting code & software for further information.

Data

Policy information about availability of data

All manuscripts must include a data availability statement. This statement should provide the following information, where applicable:

- Accession codes, unique identifiers, or web links for publicly available datasets
- A list of figures that have associated raw data
- A description of any restrictions on data availability

FACS plots show raw data.
All the raw data are available upon the request.
Mass-spectrometry data is available on ProteomXchange and single-cell sequencing data will be deposited prior to publication.
Accession codes for the single-cell RNAseq will be provided in the manuscript upon their deposition.

Field-specific reporting

Please select the one below that is the best fit for your research. If you are not sure, read the appropriate sections before making your selection.

[x] Life sciences   [ ] Behavioural & social sciences   [ ] Ecological, evolutionary & environmental sciences

For a reference copy of the document with all sections, see nature.com/documents/nr-reporting-summary-flat.pdf

Life sciences study design

All studies must disclose on these points even when the disclosure is negative.

Sample size	For human studies, sample size was determined by the availability of samples.<br>For mouse studies, sample size was >3 as per typical experimental design, and mouse availability.
Data exclusions	No data were excluded
Replication	Mouse experiments were performed at least twice. Experiments with human PBMCs could not be replicated due to limited PBMC numbers. These are rare and precious samples and so we were limited to performing all the available assays.
Randomization	Mice were assigned into experimental groups to achieve equal distribution of age and sex across experimental groups.
Blinding	Experiments were not blinded

Reporting for specific materials, systems and methods

We require information from authors about some types of materials, experimental systems and methods used in many studies. Here, indicate whether each material, system or method listed is relevant to your study. If you are not sure if a list item applies to your research, read the appropriate section before selecting a response.

Materials & experimental systems

n/a	Involved in the study
[ ]	[x] Antibodies
[ ]	[x] Eukaryotic cell lines
[x]	[ ] Palaeontology and archaeology
[ ]	[x] Animals and other organisms
[ ]	[x] Human research participants
[x]	[ ] Clinical data
[x]	[ ] Dual use research of concern

Methods

n/a	Involved in the study
[x]	[ ] ChIP-seq
[ ]	[x] Flow cytometry
[x]	[ ] MRI-based neuroimaging

Antibodies

Antibodies used	We used commercially-available antibodies as per Materials and Methods
Validation	Antibodies were validated by the manufacturers and titrated in our laboratory prior to their use

Eukaryotic cell lines

Policy information about cell lines

Cell line source(s)	C1R cells were obtained from the Department of Biochemistry and Molecular Biology & Infection and Immunity Program, Biomedicine Discovery Institute Monash University
Authentication	All cell lines were used at low passage number. C1R cells were routinely tested for HLA expression levels.
Mycoplasma contamination	Cell lines were tested using an in-house PCR kit.
Commonly misidentified lines<br>(See ICLAC register)	None used.

Animals and other organisms

Policy information about studies involving animals; ARRIVE guidelines recommended for reporting animal research

Laboratory animals	HHD-A24 mice
Wild animals	N/A
Field-collected samples	N/A
Ethics oversight	Animal experiments were approved by the University of Melbourne Animal Ethic Committee (1714108).

Note that full information on the approval of the study protocol must also be provided in the manuscript.

Human research participants

Policy information about studies involving human research participants

Population characteristics	Please refer to Supplementary Table 1
Recruitment	Samples were recruited through The University of Melbourne, Menzies school of Health research, Australian Red Cross Lifeblood, DonateLife Victoria, and The Alfred Hospital (through co-authors A Cheng and T Kotsimbos). Signed informed consents were obtained from all blood donors prior to the study. Spleen, lung and lymph node samples were obtained from deceased organ donors. Spleen and lymph node samples were obtained via DonateLife Victoria. Lung samples were obtained from individuals who passed of non-immunological conditions nor influenza infection. Tonsils were obtained from healthy individuals undergoing tonsillectomy (Launceston General Hospital,Tasmania, Australia) Is this correct?.
Ethics oversight	Human experimental work was approved by the University of Melbourne Human Ethics Committee (ID 1955465.2, 1443389.4, 1441452.1 and 1954302.1), the Australian Red Cross Lifeblood Ethics Committee (ID 2015#8), the Alfred Hospital Ethics Committee (ID 280/14), Monash Health Human Research Ethics Committee (HREC) (ID HREC/15/MonH/64, RMH local reference number 2016/196), HREC of Northern Territory Department of Health and Menzies School of Health Research (ID 2012-1928) and Tasmanian Health and Medical HREC (ID H0017479).

Note that full information on the approval of the study protocol must also be provided in the manuscript.

Flow Cytometry

Plots

Confirm that:

- [x] The axis labels state the marker and fluorochrome used (e.g. CD4-FITC).
- [x] The axis scales are clearly visible. Include numbers along axes only for bottom left plot of group (a 'group' is an analysis of identical markers).
- [x] All plots are contour plots with outliers or pseudocolor plots.
- [x] A numerical value for number of cells or percentage (with statistics) is provided.

Methodology

Sample preparation	Samples were prepared as described in the Methods
Instrument	BD LSR Fortessa was used for acquisition of data and BD FACSAria for cell sorting
Software	BD FACS Diva, FlowJo
Cell population abundance	Only single cell sorting was performed, which was confirmed by the presence of single TCR chains.
Gating strategy	Gating was performed as previously described Koutsakos et al. 2019 DOI: 10.1038/s41590-019-0320-6

- [x] Tick this box to confirm that a figure exemplifying the gating strategy is provided in the Supplementary Information.
